# Sustained Action of Developmental Ethanol Exposure on the Cortisol Response to Stress in Zebrafish Larvae and Adults

**DOI:** 10.1371/journal.pone.0124488

**Published:** 2015-04-13

**Authors:** Matteo Baiamonte, Caroline H. Brennan, Gavin P. Vinson

**Affiliations:** School of Biological and Chemical Sciences, Queen Mary University of London, London, United Kingdom; University Zürich, SWITZERLAND

## Abstract

**Background:**

Ethanol exposure during pregnancy is one of the leading causes of preventable birth defects, leading to a range of symptoms collectively known as fetal alcohol spectrum disorder. More moderate levels of prenatal ethanol exposure lead to a range of behavioural deficits including aggression, poor social interaction, poor cognitive performance and increased likelihood of addiction in later life. Current theories suggest that adaptation in the hypothalamo-pituitary-adrenal (HPA) axis and neuroendocrine systems contributes to mood alterations underlying behavioural deficits and vulnerability to addiction. In using zebrafish (*Danio rerio*), the aim is to determine whether developmental ethanol exposure provokes changes in the hypothalamo-pituitary-interrenal (HPI) axis (the teleost equivalent of the HPA), as it does in mammalian models, therefore opening the possibilities of using zebrafish to elucidate the mechanisms involved, and to test novel therapeutics to alleviate deleterious symptoms.

**Results and Conclusions:**

The results showed that developmental exposure to ambient ethanol, 20mM-50mM 1-9 days post fertilisation, had immediate effects on the HPI, markedly reducing the cortisol response to air exposure stress, as measured by whole body cortisol content. This effect was sustained in adults 6 months later. Morphology, growth and locomotor activity of the animals were unaffected, suggesting a specific action of ethanol on the HPI. In this respect the data are consistent with mammalian results, although they contrast with the higher corticosteroid stress response reported in rats after developmental ethanol exposure. The mechanisms that underlie the specific sensitivity of the HPI to ethanol require elucidation.

## Introduction

In humans and other mammals, the hypothalamo-pituitary-adrenal (HPA) axis may have a crucial role in the physiological and behavioural response to addictive agents, including alcohol, and in the processes involved in withdrawal and reinstatement [1]. Acutely, alcohol treatment generally enhances corticosteroid secretion in rats [2, 3] and in human subjects, probably as a consequence of increased ACTH secretion [4, 5]. This in turn is a response to enhanced CRF and AVP production and release from the PVN [6–8]. On the other hand, binge-exposure and chronic alcohol abuse blunt these actions, leading to depressed HPA activity and corticosteroid secretion [9]. Both in humans and in animal models, alcohol withdrawal may then lead to enhanced plasma (or salivary) corticosteroid [10–13], and, in rats, more prolonged corticosteroid elevation in the brain [13]. Elevated corticosteroid may in turn promote relapse [14], indeed some authors consider corticosteroid to be an essential component of behavioural responses to alcohol and other drugs [15–17]

In development, exposure to ethanol and other addictive substances has been associated with later susceptibility to behavioural deficits, including addiction. These form part of a spectrum of deleterious effects, known as Fetal Alcohol Spectrum Disorder (FASD) [18].

It is unclear to what extent the sequelae of developmental alcohol exposure are attributable to its actions on the HPA axis, though in mammals much evidence suggests the HPA is significantly perturbed. Thus both male and female rats that have been developmentally exposed to ethanol show a greater increase in plasma ACTH and corticosterone after stress, though baseline levels are usually unchanged [19–24].

The value of the zebrafish as a model for the study of the behavioural effects of ethanol has been clearly demonstrated [25–28], and the effects on behaviour of ethanol exposure during development reflect to a degree findings obtained in mammals [29].

To understand the role of the zebrafish hypothalamus-pituitary-interrenal (HPI) axis (the homologue of the mammalian HPA) in such responses, the present study set out to determine the effects of developmental exposure to ethanol on subsequent cortisol levels and responses to stress.

## Materials and Methods

### Animal maintenance

All animal work was carried out following approval from the Queen Mary Research Ethics Committee, and under licence from the Animals (Scientific Procedures) Act 1986. Care was taken to minimize the numbers of animals used in this experiment in accordance with the ARRIVE guidelines (http://www.nc3rs.org.uk/page.asp?id=1357. Fish were bred and reared in the aquarium facility at Queen Mary University of London, licenced by the UK Home Office. Zebrafish (*Danio rerio*) adults from the Tuebingen wild type (TUWT) line were kept in glass breeding tanks in fish water that contain sodium bicarbonate (0.9mM), calcium sulphate (0.05mM) and marine salts (Sigma, Poole, UK; 0.018g/l). Fish were maintained on a constant 14h light: 10h dark cycle at 28°C. They were fed 3 times a day with Zmsystems ZM-000 high protein food particle (Tecniplast UK. London) from 5dpf-10dpf, ZM-100 and paramecium from 11dpf-14dpf, and, ZM-200 and brineshrimp from 14dpf-30dpf. At one month of age, animals were transferred into the aquaria where they were fed Zmsystems flake food and brineshrimp.

### Embryo spawning

Fertilised eggs were collected by natural spawning. At 24h post fertilisation (pf) morphological criteria such as the head-trunk angle and the optic vesicle length [30] were used to evaluate their embryonic stage. Embryos were distributed into groups of 50 in 40ml aquarium water in petri dishes then reared in an incubator at 28°C. Ethanol was added as required.

### Larval size assessment

Larval size at 9dpf was determined using eLaborant, an automated image detection software made by eLaborant (Leiden, Netherlands). The method involves tracing a virtual line around the image of the animal, estimating both main axis length and the pixels contained within traced line perimeter.

Tissue dry weight was obtained by homogenizing 25 larvae in 1.5mL pre-weighed microcentrifuge tubes, using 500μl of ice-cold 3.5% v/v perchloric acid with a microcentrifuge tube pestle on ice. Samples were then evaporated in a Univapo 100H speed vac for 1 hour, using a Unijet II aspirator vacuum pump. Microcentrifuge tubes were weighed again using a precision analytical Sartorius 2006 MP scale, to obtain the dry weight of the samples.

Control animals or those treated with 20mM and 50mM ethanol solutions were photographed in high resolution (4064 x 4064) using a Nikon D800 camera. The software analysed 60 larvae in each group and provided the pixel count/ central axis length per animal.

### Alcohol treatment and uptake.

For developmental ethanol exposure, treated larvae were exposed from 1-9dpf to 20mM and 50mM GPR ethanol (VWR, Lutterworth UK). Controls were handled similarly, but ethanol was omitted.

To verify ethanol uptake, ethanol concentrations in embryonic and larval tissue were assessed using an alcohol dehydrogenase based spectrophotometric method adapted from Reimers et al [31].

After treatment with ethanol, 25 live embryos or larvae (with intact chorions if applicable) were transferred to 1.5 ml microcentrifuge tubes (Starlab, Milton Keynes, UK) on ice. Animals were quickly rinsed twice with 500μl of ice-cold distilled water and homogenized with a microcentrifuge tube pestle in 3.5% v/v perchloric acid (500μl, Sigma).

Samples were centrifuged at 4°C for 10 minutes at 12,000g and then stored in paraffin sealed tubes at 4°C until all samples were collected, or placed on ice and immediately used. Standard curves were constructed using six ethanol standards, ranging from 100mM to 3.125mM, yielding a non-linear quadratic polynomial function (*r* ^*2*^ = 0.99). Two replicates for each standard and samples were produced. The initial reaction mixture was 870μl of NAD+ (Sigma; 1mg/ml in 0.5M Tris, pH 8.8) with 43.5μl of the standard or the sample (in perchloric acid) in 1.5ml microcentrifuge tubes.

To start the reaction, 86.5μl ADH (Sigma; 0.75mg/ml in water) was added to the microcentrifuge tube, the cap was closed, and the content mixed and incubated at 37^o^ for 10 min then transferred to a Starlab 1.5ml semimicro cuvette. NADH production was evaluated from its absorption at 340nm. A blank reaction substituting 3.5% v/v perchloric acid for the ethanol solutions for was used to calibrate the spectrophotometer initially.

### Stress tests: Air exposure and freezing

For the air exposure challenge, 9dpf larvae were transferred into 6-well plates wells mounted with sieve inserts. Wells were filled with 10ml of fish water. Larvae were grouped 12 animals per well, and left to habituate for 1 hour in a lit and silent environment. Sieve inserts were lifted and placed in paper towels for 1 min, air exposing the subjects inside the sieve, then immediately replaced in the wells. Control animals remained in the wells.

Animals were then quickly pipetted into 1.5ml microcentrifuge tubes, water was removed, and tubes were immediately flash frozen in liquid nitrogen and stored at -80^o^.

Adult fish were individually netted from pair-housed tanks into white opaque tanks (42.5cm length x 16cm width x 17.5cm height) containing fish water (2l). These tanks contained a smaller clear acrylic bottom-perforated tank insert (Aquatic Habitats, Apopka, USA), to allow easy and quick air exposure of the animals.

Tanks were covered with an opaque lid, and fish were left to habituate for 1h in a lit and silent environment. Preliminary experiments indicated that, in contrast to the larvae, there was no cortisol response when the adults were air exposed for 1 min, but there was a good response after just 30s (see Results). Accordingly, experimental animals were routinely air exposed by lifting the small acrylic tank for 30s, before placing in the larger opaque tank.

After 6 min animals were transferred into fish water at 0°, dried on a paper towel, weighed and placed into 7ml polystyrene sample containers (Sterilin, Newport Gwent, UK), flash frozen in liquid nitrogen, and stored at -80^o^.

### Larval whole body cortisol extraction

A modified version of the protocol of Alderman et al. [32] was used for homogenization and extraction. Fish were thawed in microcentrifuge tubes on ice and homogenized in 200μl of ice-cold 1x phosphate buffered saline (PBS, Sigma) for 10s using a Sonoplus UW2070 ultrasonicator (Bandelin, Berlin, Germany). PBS (200μl), was used to rinse the equipment’s needle, and collected into the microcentrifuge tube. Aliquots (50μl) were withdrawn for protein quantification.

Cortisol was extracted into 500μl ethyl acetate (Fischer Chemical, Loughborough, UK). Samples were vortexed for 30 seconds, centrifuged at 5000rpm for 10 min and frozen at -80C^o^. The organic layer was then decanted into 12ml glass screw-top vials (VWR, Lutterworth, UK). The extraction was repeated twice more.

Tubes containing the combined ethyl acetate extracts were placed in a waterbath set at 60^°^C and the ethyl acetate evaporated under a stream of nitrogen. PBS (200μl) was added to the tubes, vortexed for 30 seconds, and kept at -20^o^ C until assessed using a human salivary cortisol kit (Salimetrics Newmarket, UK).

### Adult whole body cortisol extraction

Fish were thawed on ice in the 7ml Sterilin containers, then weighed and homogenised in 2 X PBS (w/v). The rotor blade was washed with an equal volume of PBS (i.e. 2 X BW w/v), which was then added to the homogenate.

Ethyl acetate extraction was performed as above. However, in the extracts of adult fish, an excess of lipid was precipitated on the vessel wall after evaporation. To prevent any interference with the assay, this was eliminated by partitioning the dried extracts between 500μl PBS and 500μl hexane (BDH, Poole, UK), and the organic layer was discarded. The aqueous phase was stored at -20^o^ C until required for assay. Recoveries of authentic cortisol added to tissue homogenates and extracted by this procedure was ~100%.

### Protein quantification

Tissue protein content was assayed using the Bradford reagent (Bio-Rad, Hemel Hempstead, UK) according to the manufacturers’ protocol, using a bovine serum albumin standard curve.

### Cortisol assay

Samples were thawed on ice and 50μl used for assay, performed according to the manufacture’s specifications.

Adult whole body cortisol values were normalised against body weight, and larval and juvenile cortisol values were normalized against tissue protein content.

The Salimetrics human salivary kit was validated for zebrafish whole body cortisol use. The minimum cortisol value that gave readings significantly different from zero was 0.12ng/sample. Both inter- and intra-assay coefficients of variation obtained from replicate assays of tissue extracts were less than 6%. When authentic cortisol (3ng) was added to tissue homogenates and assay values compared with identical samples without addition, recovery was between 101% and 104% and the correlation between the two sets of data gave r^2^ = 0.94, (*P*<0.0001). Specificity data provided by Salimetrics show negligible cross-reactivity with a range of possible contaminating steroids.

### Statistics

The dependent variables in the different experiments were, respectively, tissue ethanol concentration, animal size, weight, and whole-body cortisol concentration. These were assessed in the various treatment groups, viz. handling control and ethanol exposure at different concentrations. ANOVA and Student t-tests were used to assess statistical significance. The tests were evaluated with respect to type-1 error rate of 0.05.

## Results

### Ethanol uptake

**Fig 1:**
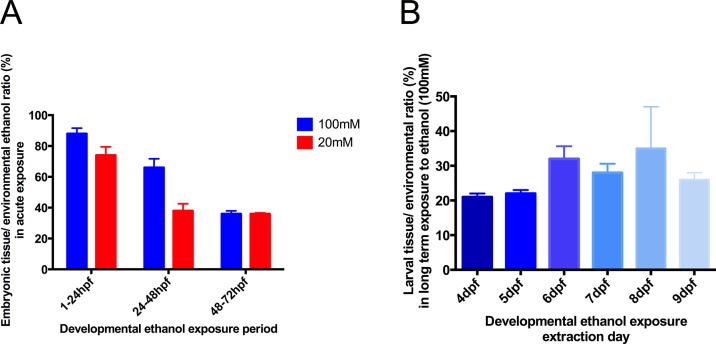
Assessment of embryonic tissue ethanol concentration compared to waterbath ethanol concentration following acute 24-hour ethanol exposure of zebrafish embryos. **A** Alcohol was assayed in whole embryos developmentally exposed to an ethanol waterbath concentration of 100mM and 20mM for a period of 24 hours, using 1-24hpf, 24-48hpf and 48-72hpf periods. **B** Tissue ethanol content after exposure to 100mM ethanol during the whole period 1-9dpf. Values are means ± SE, 3 samples collected on different days were used for each time period, 25 animals per sample. Values for tissue ethanol in the absence of added ambient ethanol were less than 1% of those illustrated, and not significantly different from zero.

Zebrafish embryos acutely exposed to ethanol for 24 hours showed differences in subsequent tissue ethanol concentration depending on their developmental stage (Fig 1A).

At 1-24hpf, tissue concentrations approached those in the ambient water, but at 24-48hpf, tissue values were significantly lower, and at 48-72hpf ethanol concentrations stabilised at 36% of the ambient ethanol concentrations of both 20mM and 100mM. During chronic treatment from 3-9dpf, tissue ethanol content never significantly exceeded these values (Fig 1B) and the tissue/ambient concentrations can therefore be assumed to be in linear relationship. The ambient concentrations used in subsequent experiments of 20mM and 50mM were those calculated to deliver tissue concentrations to reflect moderate alcohol use in humans. Maximally, tissue ethanol reached about 40μg/mg dry weight.

### Animal size

**Fig 2:**
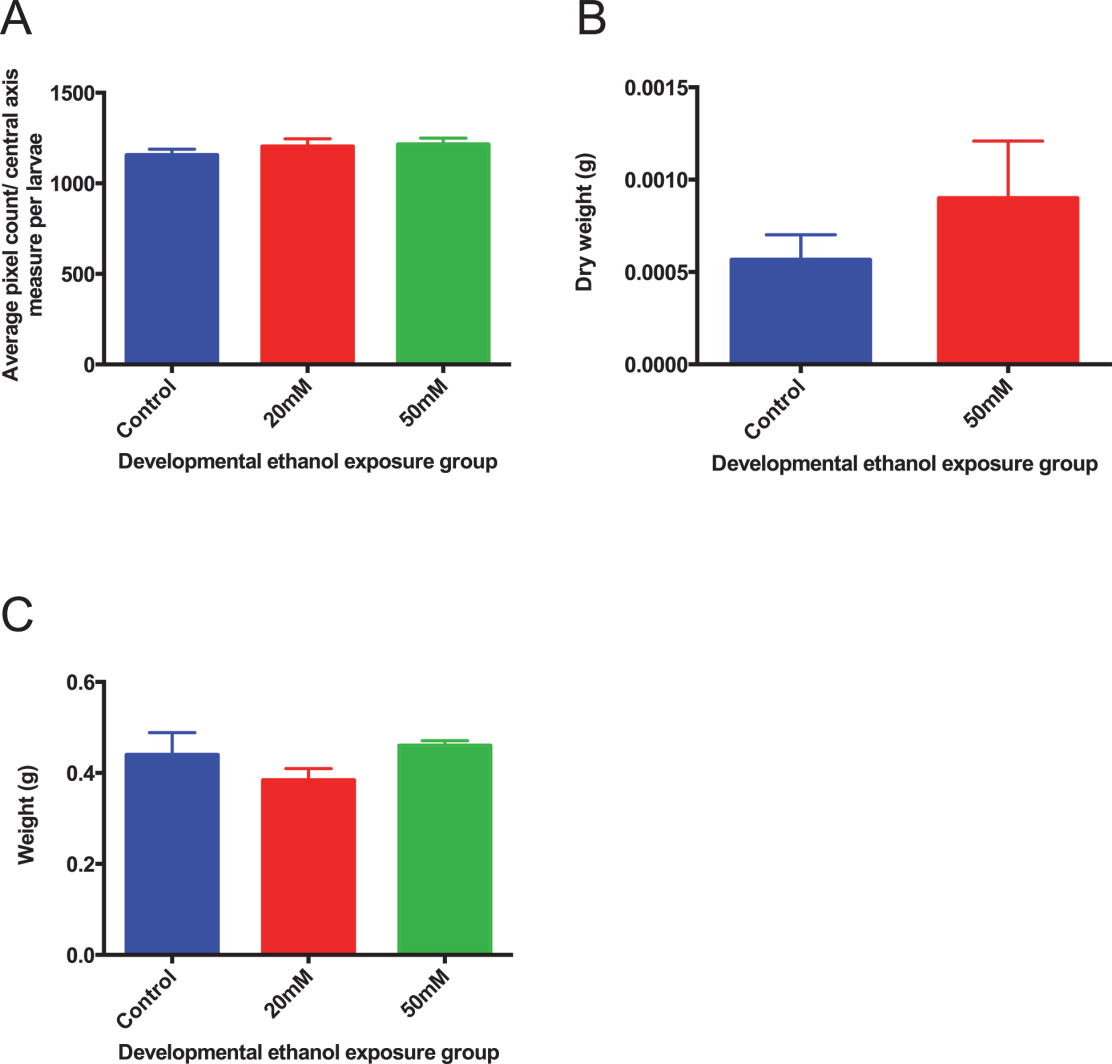
Effects of ethanol exposure from 1-9dpf on larval development A. size at 9dpf measured using eLaborant software. **B** dry weight at 9dpf. **C** body weight at 6 months. There were no differences between groups. Values are means ± SE, 3 samples were used per group, with 20 (A) or 25 (B) animals per sample.

Neither larval size nor dry body weight were affected by alcohol treatment from 1-9dpf, nor was body weight at 6 months (Fig 2). No animals died during the course of ethanol treatment, subsequent survival was similar in treated and control animals.

Visually, the treated animals had no craniofacial deformity or any body oedema compared to controls, and appeared normal, with normal locomotor activity.

### Tissue cortisol and the effects of stress

**Fig 3:**
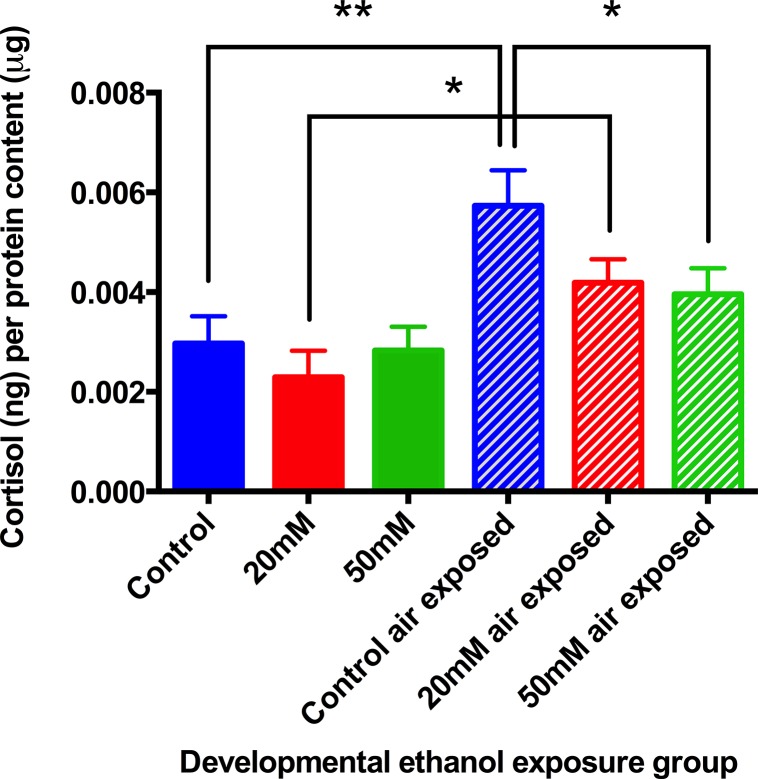
Stress-reactivity in 9dpf larvae measured by whole-body cortisol concentration. Experimental animals were developmentally treated with ethanol from 1dpf-9dpf with 20mM and 50mM ethanol concentrations. At 9dpf animals were either flash frozen immediately or air exposed then frozen. Air stressed animals showed decreased tissue cortisol concentrations with increasing ethanol concentration exposure during development. Means ± SE, 6 batches of animals per group, *P< 0.05, **P<0.01, by ANOVA and “t” test.

In larvae at 9dpf, there were no differences in cortisol between unstressed control and unstressed ethanol treated animals. However, after air-exposure stress, the increase in cortisol seen in the animals previously exposed to both 20mM and 50mM ethanol was significantly lower than controls (Fig 3), moreover, whereas cortisol in the 20mM group was still increased by stress, cortisol in the 50mM group was not significantly different either from the unstressed controls, or the unstressed 50mM group.

**Fig 4:**
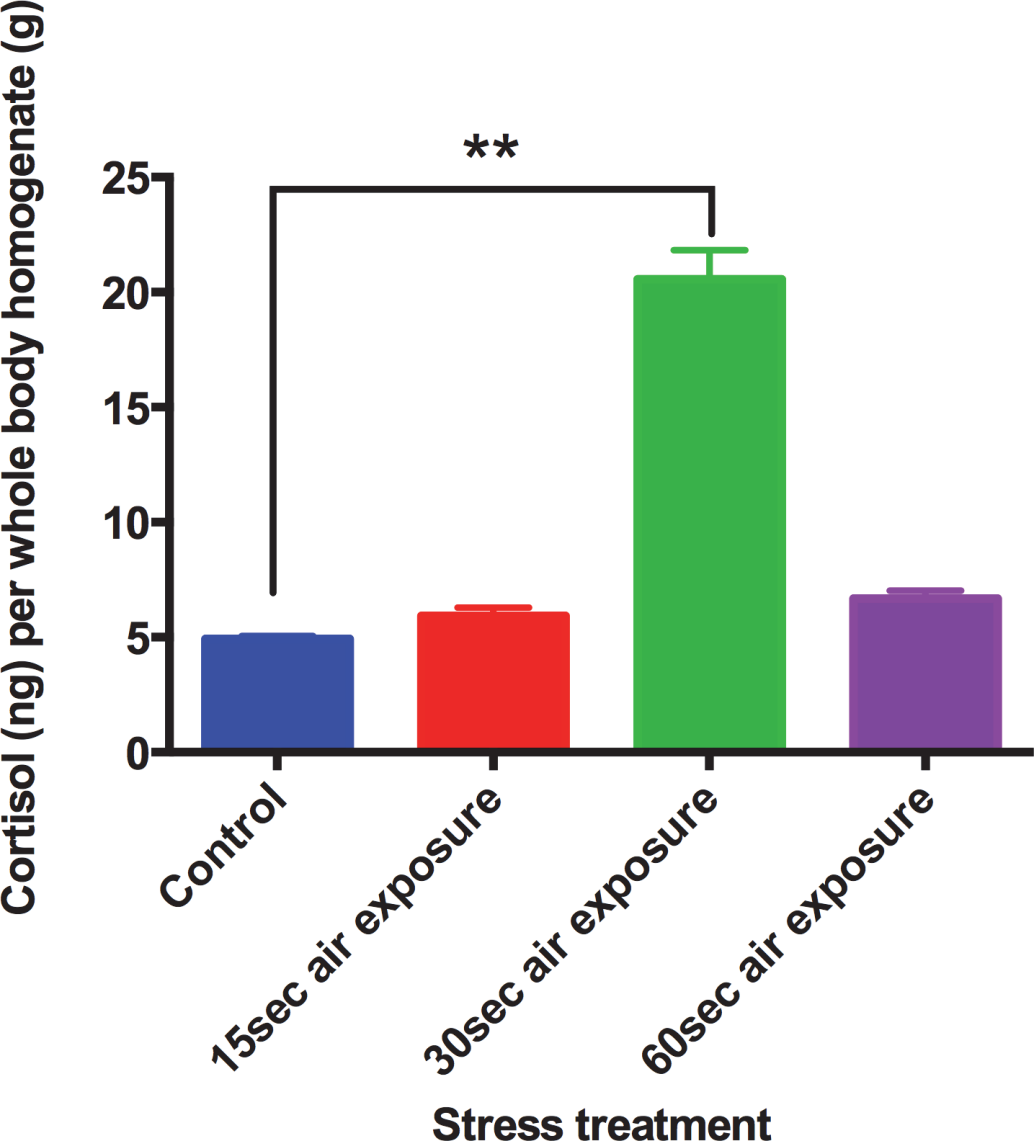
Stress-reactivity measured by whole-body cortisol concentration of zebrafish 6-month-old adults. Animals were either flash frozen or stressed, either by air exposure at a specific time or acute exposure to ethanol (1%), followed by freezing 6 minutes later. Zebrafish adults showed increased cortisol concentration when air exposed for 30s, but not after 60s exposure. Means ± SE, 3 batches (on different days) of 3 samples of 10 animals were used. *P*<0.01, ANOVA <0.01).

Six month old adult zebrafish were used in a pilot study to verify the effects of air stress on cortisol concentration treatment. Whole-body cortisol concentration following 1min air exposure was not different from that obtained from unstressed control animals (Fig 4). A 30s air exposure yielded a significant increase in tissue cortisol concentration (Fig 4) and therefore a 30s air stress was adopted as standard procedure.

**Fig 5:**
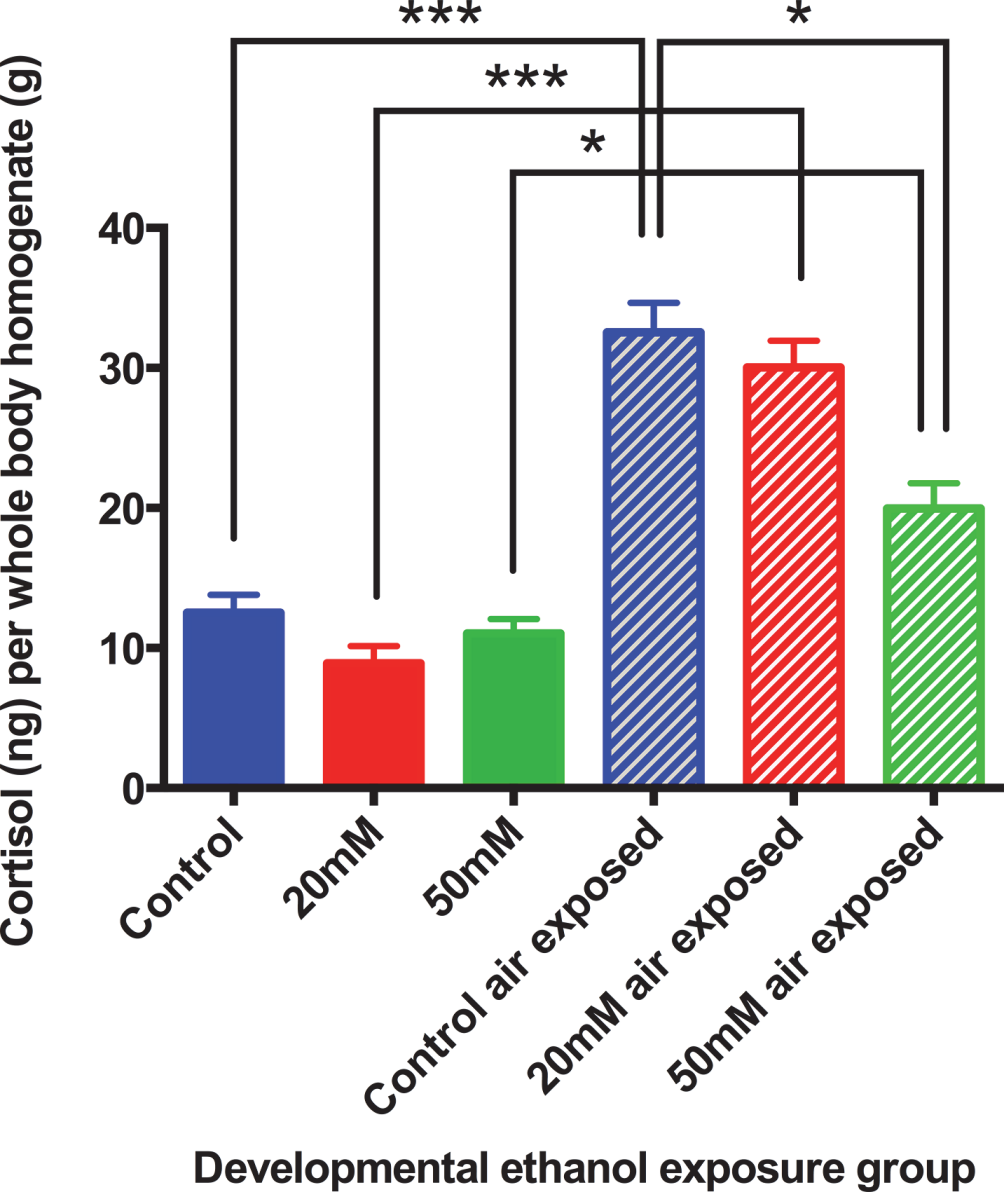
Stress-reactivity measured by whole-body cortisol concentration of zebrafish 6-month-old adults. Treated animals were developmentally exposed to ethanol from 1dpf-9dpf with 20mM or 50mM ethanol concentrations. At 6 months animals were either flash frozen immediately or air exposed and frozen 6 minutes later. Zebrafish adults showed decreased cortisol content with increasing ethanol concentration exposure during development. Means ± SE, 9 batches of animals per group. *P<0.05 **P<0.01 “t” test and ANOVA.

There were no differences in body cortisol content between control animals and those previously treated with ethanol when unstressed. Air stress provoked a significant enhancement of body cortisol in the control animals and in the 20mM ethanol group, and these two responses were similar. However, animals that had been treated with 50mM ethanol showed a significantly attenuated response to the stress compared with the stressed controls, although the cortisol values were nevertheless greater than in the unstressed 50mM group (Fig 5).

## Discussion

The impact of exposure to alcohol during mammalian development is known to be profound, and may lead to a wide range of structural, physiological and behavioural dysfunctions, culminating in humans in FASD. These are incompletely understood [18].

To further our understanding, a simpler and more tractable animal model has been sought in the zebrafish, already widely used in many aspects of normal physiology and in disease, as well as for drug screening. In this context several recent papers have examined the effects of developmental ethanol exposure on zebrafish behaviour (e.g. refs.[29,33,34]), invariably drawing attention to the parallels with mammalian species, including the human.

In mammals, the HPA axis has been implicated in the processes involved in the development of addiction and relapse, though its possible roles and the mechanisms involved remain obscure [1,5]. The aim of the present study was therefore to extend the study of developmental ethanol exposure to its effects on the stress response of the zebrafish HPI axis.

The ethanol concentrations used were selected by first assessing body uptake of ethanol from environmental water. Before 48hpf the embryo was unprotected from ethanol penetration (Fig 1), but subsequently tissue concentrations were only a fixed fraction of the ambient ethanol concentration. This fraction, 36%, was the same at ambient concentrations of both 20mM and 100mM, and it can be concluded that the ambient/tissue ethanol content is linearly related. Following this assessment, the experimental concentrations used of 20mM and 50mM were those calculated on this basis to deliver tissue concentrations that could reflect those occurring in moderate alcohol use in humans, maximal tissue ethanol concentrations were about 40μg/mg tissue.

Fish exposed to ethanol from 1-9dpf showed no difference from controls in size or body weight (Fig 2) or locomotor activity (not shown) at the end of this period. These results are consistent with the literature, and for example in the study by Reimer et al [31], exposure to up to 150mM ethanol from 3-48hpf or 3-24hpf gave no changes in development or morphology, either at 5dpf or in the 6 month adults.

While there may be various mechanisms that affect circulating cortisol levels at the margin, only one is paramount, that is the integrated function of the HPA or HPI axis. It is circulating cortisol itself, and cortisol alone that reflects the totality of HPA(I) function. It is for this reason that circulating cortisol is almost universally used as the only reliable measure of stress responses, in fish as well as in mammals (e.g. refs [35–38]). Collection of plasma is not feasible in studies of acute stress in zebrafish, and therefore whole body cortisol content was adopted as a reasonable alternative, perhaps comparable to plasma data from rodents and humans. Others have made the same choice [39]. While tissue values may not necessarily reflect rates of secretion, in mice the evidence suggests that (with some exceptions) the two are largely related [40]. Nevertheless, it is always possible that local steroid metabolism, for example by 11β-hydroxysteroid dehydrogenase type 2, may affect local tissue cortisol concentrations, and thus behaviour, or brain corticotrophin releasing hormone and the HPA [41].

From Figs 3 and 5 it is clear that developmental exposure to ethanol had no effect on basal tissue cortisol content in either larvae or adults. What is striking is its persistent effect on the response to acute stress. There is a characteristic elevation in tissue cortisol after a short air-exposure stress—confirming the validity of using tissue cortisol as an index of HPI activity. There is a characteristic dynamic in this response, and in adults it is quite short-lived (Fig 4), necessitating sampling after just 30s stress. In both larvae and adults at 6 months, early exposure to ethanol attenuated, and in some cases actually eliminated altogether, the HPI response to stress, as shown by tissue cortisol content (Figs 3 and 5). Although the treated larvae were still maintained in ethanol when these tests were carried out, the adults had been free of ethanol since 9dpf.

Physiologically and morphologically the treated animals are indistinguishable from normal, as noted above, and this extends to the unstressed function of the HPI. It is the response to stress alone that is impaired. The mechanisms here are currently obscure, though perhaps endocrinological, caused by defective corticotrophin or corticotrophin releasing hormone function, or by impaired steroidogenesis. At larval stages, since they remained exposed to ethanol, it could possibly be associated with its anxiolytic action. However, it is the persistence of the effect into the adults that suggests that a profound change has occurred during development in the treated animals, and this must be linked to irreversible structural or functional changes, presumably in the brain. A similar persistence of the behavioural consequences of early ethanol exposure in zebrafish leads to the same general conclusion [29,33,34]. Accordingly it is now appropriate to address the mechanisms that link behavioural effects to the HPI in these animals.

It is remarkable that this is similar to the conclusions reached by others in the mammalian and indeed in the human context, in which the HPA is thought to be involved in the effects of early ethanol exposure [1,5]. There are discrepancies, in mammals developmental alcohol generally leads to a hyperresponsive HPA axis [21, 22,42–44], though not invariably [23,24].

Nevertheless, as in mammals, it does seem likely that the zebrafish HPI is particularly susceptible to ethanol during development. Further study of its mechanism of action may yet throw light on what in mammals is so difficult to understand.
