# Correction: Sustained Action of Developmental Ethanol Exposure on the Cortisol Response to Stress in Zebrafish Larvae and Adults

**DOI:** 10.1371/journal.pone.0128050

**Published:** 2015-05-08

**Authors:** Matteo Baiamonte, Caroline H. Brennan, Gavin P. Vinson

The following information is missing from the Funding section: Caroline Brennan was supported by (UK) Medical Research Council grant G1000053.
